# Therapeutical and Pharmaceutical Notes

**Published:** 1901-12

**Authors:** W. J. Martin

**Affiliations:** Kankakee, Ill.


### THERAPEUTICAL AND PHARMACEUTICAL NOTES.

UNDER THE DIRECTION OF W. J. MARTIN, M.D.C.,
KANKAKEE, ILL.

PRODUCTION OF BALSAM PERU. Preuss described at the meeting of the German Pharmaceutical Society the method employed in the extraction of balsam Peru from the tree. The balsam tree grows wild in San Salvador, sometimes isolated and sometimes in groups. There do not appear to be any special plantations of it, but its arrangements in rows suggests that some trees at least were planted. The tree is from twenty metres to twenty-five metres high, rarely reaching thirty metres. The balsam owes its typical qualities neither to the wood nor to the bark, as such, but to the treatment to which the tree is subjected. It undergoes a process of wounding and bruising with an axe or stone. Pieces of bark are shredded down, laying open the wounded patch, when, after a few days, the balsam trickles out and is caught. After this the wounded place is heated with torches, more balsam exudes, and deep notches are made. Finally it is reheated, and the remainder of the balsam is obtained. Then the worker scratches off the inner bark down to the wood, breaks it up to powder, and boils it out with water, which, after separation and pressing, gives the "back balsam," which is more concentrated than the first obtained, and is of a stronger odor. The balsam Peru of trade is a mixture of the two in definite proportions; 100 trees yield 300 to 500 pounds of balsam. It is not likely that balsam Peru is locally adulterated by the merchants, since it is so cheap.—*Bulletin of Pharmacy.*

VILLATE'S SOLUTION. ℞.—Solution of subacetate of lead, half fl. oz.; sulphate of copper, 1 Troy oz.; sulphate of zinc, 1 Troy oz.; diluted acetic acid, U. S. P., 13 fl. oz.; dissolve the sulphate of copper and sulphate of zinc in the diluted acetic acid, add the solution of subacetate of lead, and agitate thoroughly; set the mixture aside, so that the precipitate may subside. Then decant or siphon off the clear liquid, and preserve it for use.

This is an astringent and escharotic solution, and may be usefully employed for injecting into fistulous tracts and sinuses, such as fistulous withers, poll evil, quittor, indolent skin abrasions, etc. It is largely employed in French veterinary practice.

Unusual Number of Tapeworms in a Dog. H. Taylor, M.R.C.V.S., *Veterinary Journal* for Ootober, 1901, records the following case: Last month we had brought to the college an Airedale terrier in good condition, and, to all appearances, in very good health, to undergo treatment for worms. This was proceeded with by fasting the dog for forty-eight hours, and administering 35 minims of tenaline, in water, that dose corresponding to his weight. In twenty minutes he evacuated a quantity of feces and a tangled mass of tapeworms, which, on being teased out and counted, was found to be composed of sixty-six tænia. The worms were, on an average, about 75 mm. long, and they all appeared to be of one variety, viz., tænia serrata. Neumann (*Parasites and Parasitic Diseases of the Domesticated Animals*) says for tænia serrata the number of individuals which Bertholus and Chauveau found in the dog varied from one up to sixty-four, so we may take the above number as exceptional. A day or two after the dog was given another dose of tenaline, but we did not succeed in augmenting our previous number.

A Royal Commission has been appointed in Great Britain to study the relations of bovine and human tuberculosis. The commission consists of Sir Michael Foster, Dr. Sims Woodhead, Dr. Harris Cox Martin, Prof. J. McFadyean, and Prof. R. W. Boyce.

Equine Diseases in the Prussian Army. In the *Veterinary Sanitary Report of the Prussian Army for the Year 1900* some interesting statistics are given of the various diseases affecting army horses; the total number of horses treated was 30,399, about 95 per cent. of these were saved by medical skill and care; 1618 horses were treated for pleuro-pneumonia, as against 2301 in the preceding year and 3265 in 1898; of 1618 cases treated, 1498 were cured, 71 were destroyed; 49 remained under treatment at the end of 1900.

By far the most interesting part of the report to the practitioner is the number of cases of colic treated, because it was in this disease that by far the largest number of fatalities occurred. During 1900, 3746 cases of colic were treated; of this number, 528 died —a large mortality, it would appear, to the private practitioner. Among the exciting causes of this disease are mentioned the eating of wet and uncleaned bedding, the eating of affected food or fodder covered with hoar-frost, exposure to cold, licking up sand, eating mouldy bread, and lack of proper exercise—all causes which, with a small amount of foresight and judgment, it would appear might have been easily averted in an organization so thoroughly under discipline as an army is supposed to be.

In the treatment of pleuro-pneumonia among horses the report states that the method of immediately isolating the sick from the well was found to be beneficial and the best plan to pursue, together with a thorough cleaning and disinfecting of the premises occupied by the sick animals.

Protective inoculation of well animals against the disease was generally considered a failure. Of the 300 treated, but two died, a splendid showing for the management of this disease.

Fifty-three horses were treated for tetanus, of which number thirty-four died ; 596 cases were treated for harness and saddle-galls ; the treatment consisted in the application of, first, cooling appliances, then moist heat, Priessnitz bandages, massage, resolvents ; 4039 cases of inflammation of the tendons and ligaments were treated in the following manner : in recent cases the cooling method, cold-water bandages, followed in the course of two or three days by massage, resolvent applications, and Priessnitz bandages; only when this method failed was resort had to the actual cautery.

MANUFACTURE OF OXALIC ACID. According to United States Consular reports, oxalic acid is manufactured from sawdust by more than twenty establishments in Europe. Six are in Germany, twelve in England, one in France, and one in Belgium.

HYDROGEN DIOXIDE FOR BURNS. Hydrogen dioxide in dilute solution of 50 per cent., applied in the form of a spray, is an excellent remedy for burns and other skin excoriations.

THE SCIENCE OF PHARMACY was widely known and practised by the ancient Egyptians. Recent archæological excavations have brought to light papyrus written about 4000 years B. C., which contains explicit directions for the preparation of medical formulæ or prescriptions.

